# An algorithm for the determination and quantification of components of nucleic acid mixtures based on single sequencing reactions

**DOI:** 10.1186/1471-2105-6-281

**Published:** 2005-11-29

**Authors:** Alexander Pozhitkov, Kathryn Stemshorn, Diethard Tautz

**Affiliations:** 1 Institut für Genetik, der Universität zu Köln, Zülpicherstrasse 47, 50674 Köln, Germany; 2 Civil and Environmental Engineering, University of Washington, Seattle, 98195, WA, USA

## Abstract

**Background:**

Determination and quantification of nucleic acid components in a mixture is usually accomplished by microarray approaches, where the mixtures are hybridized against specific probes. As an alternative, we propose here that a single sequencing reaction from a mixture of nucleic acids holds enough information to potentially distinguish the different components, provided it is known which components can occur in the mixture.

**Results:**

We describe an algorithm that is based on a set of linear equations which can be solved when the sequencing profiles of the individual components are known and when the number of sequenced nucleotides is larger than the number of components in the mixture. We have implemented the procedure for one type of sequencing approach, pyrosequencing, which produces a stepwise output of peaks that is particularly suitable for the procedure. As an example we use signature sequences from ribosomal RNA to distinguish and quantify several different species in a mixture. Using simulations, we show that the procedure may also be applicable for dideoxy sequencing on capillary sequencers, requiring only some instrument specific adaptations of protocols and software.

**Conclusion:**

The parallel sequencing approach described here may become a simple and cheap alternative to microarray experiments which aim at routine re-determination and quantification of known nucleic acid components from environmental samples or tissue samples.

## Background

The identification of individual components from a mixture of nucleic acid sequences relies currently on molecular hybridization approaches, such as microarrays. Technically, these make use of the inherent combinatorial complexity of strings of nucleotides, as well as the base pairing reaction between single stranded molecules. These allow to potentially resolve vast numbers of different sequences, provided one knows which sequences can occur in the mixture. In contrast, DNA sequencing reactions do not require a priori knowledge of the sequence that is to be determined, but they can only identify one sequence at a time. However, the patterns produced in sequencing reactions have also an inherent combinatorial complexity with respect to the order and height of peaks. Accordingly, mixtures of sequencing reactions should also produce characteristic patterns which reflect the components of the mixture. Thus, it should be possible to deconvolute a mixture pattern into its individual components, provided the components that can potentially occur in the mixture are known. We propose here an algorithm that is based on solving a system of linear equations which describe the peak patterns that can theoretically be obtained. The number of sequences that can be resolved with this algorithm depends on the number of sequencing steps that can be recorded. Thus, the procedure can not resolve the very large number of sequences that can potentially be resolved by microarrays, but applications where this high resolution potential is not necessary could be done more efficiently with the parallel sequencing procedure described here.

One particular application, which we use here to exemplify the procedure, would be species identification through DNA taxonomy [1-5]. It has been proposed that the identification of organisms could be based on short characteristic strings of nucleotides from universally occurring genes, such as the mitochondrial cytochrome oxidase gene [3] or ribosomal RNAs [5]. This requires on the one hand to build a database of such sequences and on the other hand to re-sequence given samples and to compare them with the database. The re-sequencing could become more efficient, when several samples could be pooled and determined in a single sequencing reaction. Also, environmental samples of small organisms are usually obtained as mixtures and it would be highly advantageous, if the species within them could be determined without separation. We use here the example of ribosomal RNA signature sequences to show that this approach is indeed promising. For this we employed the pyrosequencing procedure [6], because of its inherent property to separate each sequencing step. However, using simulations, we suggest that dideoxy sequencing procedures [7] might work as well, provided some technical adjustments can be done.

## Results

Conventional dideoxy sequencing procedures require single templates from which labeled DNA fragments are produced, which are then resolved by electrophoresis [7]. In the pyrosequencing procedure, each individual nucleotide in a template sequence is probed for incorporation of all possible nucleotides and a light signal is generated when the incorporation is successful [6]. In both procedures, the signal intensity depends on the concentration of the template and the sequence context of the nucleotide that is being determined. The latter depends on the enzyme that is used, as well as on the reaction conditions. However, for a given enzyme and condition, each template will produce a reproducible pattern of signals.

The system of linear equations that is used for the deconvolution algorithm (see methods) can be solved when the number of equations is at least equal to the number of components in the mix. Each equation reflects one sequencing step, i.e. the recorded sequence length determines the complexity of the mixture that can be analysed. In practice, one will require more steps than the theoretical minimum, to compensate for non-informative (i.e. conserved) positions and experimental noise. Since the pyrosequencing procedure produces a stepwise output for each nucleotide position probed, it can be directly employed to test whether the algorithm works under practical conditions.

### Implementation for pyrosequencing

**Figure 1:**
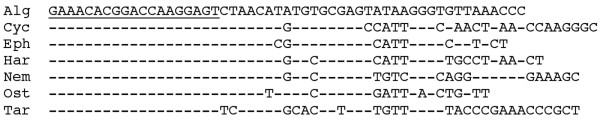
Sequence alignments for the seven taxa used in this study covering the region that is probed by the pyrosequencing procedure with 60 dispensation steps (dispensation order: A-T-G-C). The underlined part represents the primer that was used for the sequencing reaction. The length of the sequence recorded by the pyrosequencing procedure depends on the exact order of the nucleotides and the order of the dispensation steps. Hence, it is slightly different for the different sequences.

**Figure 2:**
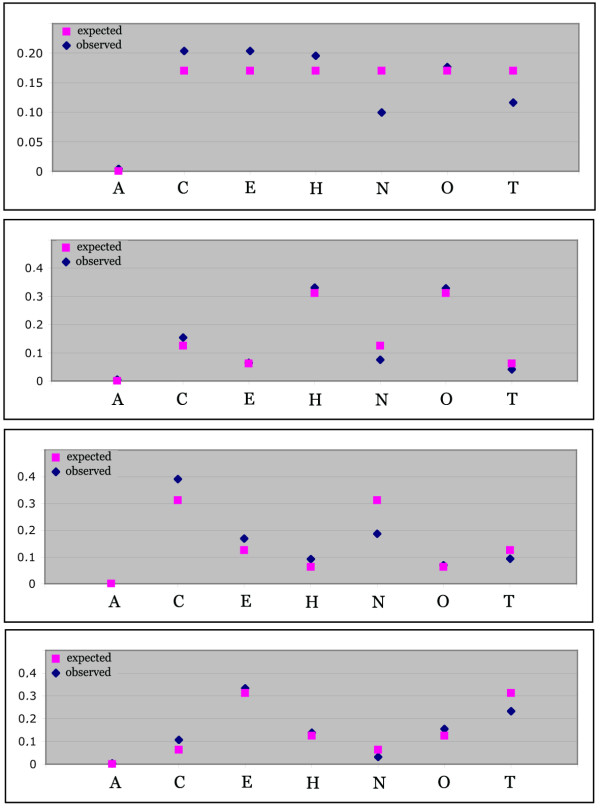
Results from the mixture experiments. Observed and expected values are plotted for each mixture. The observed values are averages from four replicates, each evaluated with four replicates of the library of profiles. The actual values of the replicates, as well as the standard deviations are listed in supplementary Table 1.

The ribosomal RNA sequences that were used for the test were derived from meiobenthos organisms. A special feature of rRNA sequences is the fact that they are composed of a patchwork of highly conserved and highly divergent regions. Hence it is possible to use a single primer for the sequencing reaction next to a region with high diversity which is particularly informative with respect to species discrimination. Figure 1 shows the relevant sequence alignments for the organisms used in this study. The respective fragments were previously cloned [5,8], allowing to determine the individual sequence profiles and to generate defined mixtures. Four different mixes were generated and sequenced in parallel to the single sequences to obtain the respective profiles. Each mix was done in four replicates and the solutions were calculated for each of the four libraries. The estimated standard deviations for each solution (see Methods), as well as for the replicates were low in most cases (data listed in suppl. Table 1). Figure 2 displays the observed and expected values for each mix (note that "Algae" was always used as a negative control and not added to the mix). This shows that the different components were indeed always identified, although not always at their expected concentration. The Nematode and the Tardigrade showed consistent downward biases, which are compensated by slight upward biases for the other components. The reasons for these biases are not yet clear. Still, the results demonstrate that the algorithm works in principle, although systematic under- or over-estimations of the relative concentration of the component might occur (see discussion).

### Simulation of dideoxy sequencing

Because the pyrosequencing procedure is more restricted with respect to the number of nucleotides that can be sequenced than dideoxy sequencing, it could be useful to adapt the procedure to dideoxy sequencing as well. However, the capillary sequencers that are nowadays used for resolving the DNA fragments are highly tuned towards the specific task of sequence determination, which interferes with the requirement of peak synchronization for the parallel sequencing procedure. Since this is a technical problem that can not be easily solved without interfering with the basic function of the respective instruments, we have simulated the results of dideoxy sequencing, to test the parallel sequencing procedure and to assess at the same time the influence of noise in the system.

**Figure 3:**
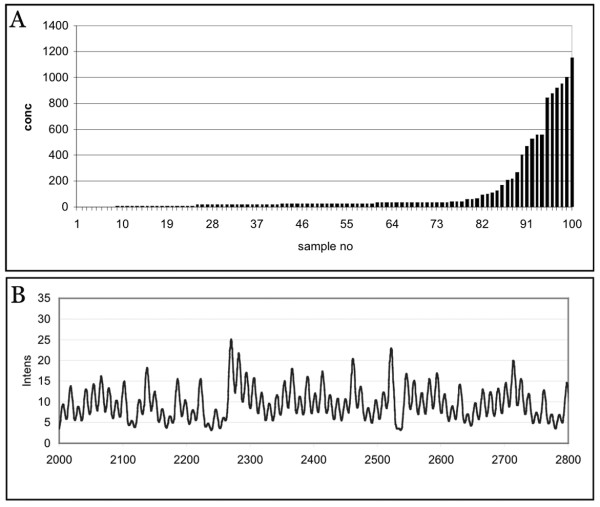
(A) Distribution of the simulated abundance profile of the components in the mix used for assessing the influence of noise in Figure 3. The concentrations were randomly assigned to 99 samples. The 0^th ^sample had concentration 0 as a negative control. (B) Section of a simulated peak profile of the mix with the abscissa depicted as a time line measured in scan numbers and the ordinate in arbitrary intensity units.

Sequencing profiles were simulated with variations of position specific peak heights (see Methods). From these profiles we generated mixtures of one hundred sequences, with concentration differences of three orders of magnitude. A typical abundance profile is shown in Figure 3 together with the simulated composite sequence profile. In the absence of noise, this profile can be unequivocally solved for each component, based on 200 sequenced positions. However, real experiments would include experimental noise. We have simulated this noise at three different levels, 1%, 5% and 10% noise in each of the peaks of the mixture. Note that the noise level is determined by the ratio of the noise to the highest peak of the electropherogram (see Methods). By calculating detection limits defined as three times standard deviation of the solution for the negative control, one can assess the level at which faithful determination is still possible. We found that for the 1% noise level, components present at approximately 0.06% or lower would not be faithfully recovered. This threshold would rise to about 0.3% at the 5% noise level and about 0.6% at the 10% noise level (Figure 3). In our experience, the actual noise levels in peak determinations for identical sequences are currently about 5% on an ABI capillary sequencer. Thus, with the currently available procedures, one would already be able to resolve a dynamic range of 30-fold concentration differences in hundreds of components.

## Discussion

Our results show that although the experimental procedures will have to be optimized, it is evident that parallel sequencing can in principle be applied to determine the components of mixtures of nucleic acids. The approach will have a particular power for applications where routine re-determination of a limited number of sequence components is required. In such cases it will be possible to experimentally determine correction factors for cases where systematic under- or over-representations of components are observed, as it was the case for two of the components in our experiments. Although we do not yet know the reasons for these deviations, it appears that they are highly reproducible, implying that correction factors would solve this problem.

The most immediate application for our procedure would be in the field of DNA taxonomy or DNA barcoding. There are currently ongoing efforts to obtain DNA barcodes for a large diversity of organisms, including those from soil [1] or benthos [5] samples. These samples include a mixture of small organisms that might be suitable indicators for ecological quality, i.e. a routine determination may become relevant for environmental monitoring. The use of ribosomal rRNA signature sequences would be particularly relevant for such samples, since the rRNA molecules occur in high concentrations in each cell, which could potentially allow using them directly as templates for the sequencing reaction. Furthermore, since the pyrosequencing procedure does not require resolving the reaction products by electrophoresis, it would seem feasible that relatively simple instruments can be constructed to determine the composite sequence profiles, even under outdoor conditions.

However, our procedure may also be useful for medical diagnostic approaches, which aim at routine determinations of limited numbers of components. It seems possible that certain diseases or cancers are characterized by the misexpression of a small set of genes. There are ongoing efforts to develop dedicated microarray assays for diagnosing these on a routine basis. Instead, one could envision using the parallel sequencing procedure to achieve the same goal. In this case one would have to preamplify the samples with a set of primers that are specific for the respective genes and which carry a universal priming site for the sequencing primer. This mixture could then be directly sequenced and the relative concentrations of the components would a measure of the level of expression in the original sample. The parallel sequencing procedure could thus at least partially substitute diagnostic microarrays in the future.

The pyrosequencing procedure that we have employed here can produce only a limited number of reliable sequencing steps, i.e. only a small number of components could be experimentally resolved. However, optimized procedures [9] can yield reading lengths of up to 200 nt, which would expand the applicability to more complex mixtures. Even longer sequence reads can be achieved with the dideoxy sequencing procedure on capillary sequencers. With these, one should be able to assess in the order of hundreds of components in parallel, depending on the level of experimental noise. The greatest problem for implementing the parallel sequencing procedure directly to currently available sequencers is the lack of synchronization between the peaks in different capillaries, i.e. the profile libraries can not be easily matched with the experimental runs which provide a complex picture of composite peaks. However, this problem may be solved by including size standards in each capillary and adjusting the recording software for peaks to these standards, a task that has to be solved in an instrument specific manner.

## Conclusion

The parallel sequencing procedure has the capacity to substitute many applications where dedicated microarrays would currently be the only solution. Although further experimental optimizations will be required, these are expected to be solvable in principle. The immediate applicability lies in the field of DNA barcoding and DNA taxonomy, but applications in the field of medical diagnostics would also seem feasible.

## Methods

### Templates and pyrosequencing

The ribosomal rRNA templates that were used for the implementation of the pyrosequencing procedure were derived from a project where the D3–D5 expansion segment region of the LSU was cloned from organisms that are present in meiobenthos samples [5,8]. The seven sequences that were chosen represent an algae (A), a nematode (N), a tardigrade (T), three crustaceans (C = cyclops, H = harpacticoid, O = ostracod) and an insect (E = ephemeroptera). The fragments were amplified from the clones using primers designed with the software PROBE [10]: forward primer 5'-GAC-CCG-TCT-TGA-AAC-ACG-G-3' and a biotinylated reverse primer 5'-ATC-GAT-TTG-CAC-GTC-AGA-A-3'. Pyrosequencing was performed with 5'-GAA-ACA-CGG-ACC-AAG-GAG-T-3' as sequencing primer according to the instructions of the supplier (Biotage, Uppsala) and on the PSQ96 MA instrument (Biotage). The pyrograms for sixty dispensation cycles were recorded and exported into Excel (Microsoft Inc.) for the further calculation steps.

#### Deconvolution of the mixture for the pyrosequencing procedure

Each component of the mixture contributes to the final pattern recorded for this mixture. Determination of the pattern contributions, i.e. quantification of the components in the mix, can be achieved by solving a system of linear equations. In theory the following system of equations describes the pyrosequencing process:

{∑i=1Nkji(Z)nji(Z)xi=Sj(Z)...
 MathType@MTEF@5@5@+=feaafiart1ev1aaatCvAUfKttLearuWrP9MDH5MBPbIqV92AaeXatLxBI9gBaebbnrfifHhDYfgasaacH8akY=wiFfYdH8Gipec8Eeeu0xXdbba9frFj0=OqFfea0dXdd9vqai=hGuQ8kuc9pgc9s8qqaq=dirpe0xb9q8qiLsFr0=vr0=vr0dc8meaabaqaciGacaGaaeqabaqabeGadaaakeaadaGabaabaeqabaWaaabCaeaacqWGRbWAdaWgaaWcbaGaemOAaOMaemyAaKgabeaakiabcIcaOiabdQfaAjabcMcaPiabd6gaUnaaBaaaleaacqWGQbGAcqWGPbqAaeqaaOWaaeWaaeaacqWGAbGwaiaawIcacaGLPaaacqWG4baEdaWgaaWcbaGaemyAaKgabeaakiabg2da9iabdofatnaaBaaaleaacqWGQbGAaeqaaOWaaeWaaeaacqWGAbGwaiaawIcacaGLPaaaaSqaaiabdMgaPjabg2da9iabigdaXaqaaiabd6eaobqdcqGHris5aaGcbaGaeiOla4IaeiOla4IaeiOla4caaiaawUhaaaaa@4F2A@

S_j _– is the peak intensity at j-th step of a specified nucleotide; k_ji_(Z) – the linear coefficient between signal intensity and incorporation event of a specified nucleotide Z (A,T,G,C) at the j-th step of sequencing for the i-th organism; n_ji_(Z) – number of available incorporation events for the nucleotide at the j-th step for the i-th organism (0,1,2,3...) since polynucleotide sequence can have successive repetitions of the same nucleotide several times; x_i _– the sought concentration of the i-th organism; N – total number of organisms.

In practice the coefficients k_ji_(Z) are unknown, hence it is necessary to record pyrosequencing profiles for each expected component prior to solving the linear system. Pre-recorded profiles represent a set of k_ji_(Z)n_ji_(Z) which is then used to deconvolute a mix. Needless to say that the dispensation order of dNTPs determines pyrograms, therefore it must be consistent throughout individual components and the sample (in our case: A-T-G-C).

In a matrix form the system can be re-written as:

N·X = S

where **N **– matrix of n_ji _multiplied by k_ji_(Z), actually the pre-recorded profiles; **X **vector of x_i_; **S **– vector of peak intensities. This system can analytically be solved by the "least squares" solution which minimizes the square of the norm of the residual difference [11]:

X = (N^**T**^·N)^**-1**^·N^**T**^·S

To obtain an estimator for the standard deviations of the solutions one has to assume that the values in the matrices N and S, being physical measurements of light intensity, are distributed normally. This assumption allows to calculate errors associated with each solution using the following procedure (according to[11]): a non-scaled covariance matrix for the vector of solutions (X) can be computed as:

*C *= **(N^**T**^·N)**^**-1**^

This matrix needs to be scaled by a factor that can be determined as follows:

sf=(NX−S)T⋅(NX−S)r−p
 MathType@MTEF@5@5@+=feaafiart1ev1aaatCvAUfKttLearuWrP9MDH5MBPbIqV92AaeXatLxBI9gBaebbnrfifHhDYfgasaacH8akY=wiFfYdH8Gipec8Eeeu0xXdbba9frFj0=OqFfea0dXdd9vqai=hGuQ8kuc9pgc9s8qqaq=dirpe0xb9q8qiLsFr0=vr0=vr0dc8meaabaqaciGacaGaaeqabaqabeGadaaakeaacqWGZbWCcqWGMbGzcqGH9aqpdaWcaaqaamaabmaabaGaeCOta4KaeCiwaGLaeyOeI0IaeC4uamfacaGLOaGaayzkaaWaaWbaaSqabeaacqWHubavaaGccqGHflY1daqadaqaaiabh6eaojabhIfayjabgkHiTiabhofatbGaayjkaiaawMcaaaqaaiabdkhaYjabgkHiTiabdchaWbaaaaa@441F@

where **X **is the solution of the above equation, *r *– number of rows and *p *– number of columns in **N **respectively [12].

The diagonal elements of the scaled covariance matrix are variances (squared standard deviations) of each solution in the vector **X**. Therefore, these diagonal elements can be used as a measure of an error associated with each solution. Note that values in the covariance matrix as well as the solution are only meaningful if there is a good correlation between **S **and **NX, **where **X **is the computed solution. Correlating **S **and **NX **allows determining how well the pyrogram of the unknown sample can be explained in terms of the pyrograms of individual components. If the correlation is poor, the design matrix **N **is not adequate to the sample (i.e. there are too many unknown RNAs in the sample) and the solution as well as covariance matrix are meaningless.

The number of steps required for an unambiguous solution must be at least as many as the number of components to be identified plus possible additional steps to provide non-singularity of the matrix **N**. Any further additional steps provide an overdefinition of the system that makes its resolution more robust. For a given set of anticipated components it is possible to determine a minimal number of sequencing steps beforehand assuming all k_ji_(Z) = 1 and n_ji_(Z) taken from the sequences of these components. A simple algorithm simulating pyrosequencing evaluates singularity of the matrix **N **at each step until **N **is no more singular. The solution of the linear equations was carried out with the MathCad (Math Soft Inc.).

#### Implementation for dideoxy sequencing

A mixture electropherogram from a capillary sequencer can be represented as follows:

y(t)=∑i=0N−1aifi(t)
 MathType@MTEF@5@5@+=feaafiart1ev1aaatCvAUfKttLearuWrP9MDH5MBPbIqV92AaeXatLxBI9gBaebbnrfifHhDYfgasaacH8akY=wiFfYdH8Gipec8Eeeu0xXdbba9frFj0=OqFfea0dXdd9vqai=hGuQ8kuc9pgc9s8qqaq=dirpe0xb9q8qiLsFr0=vr0=vr0dc8meaabaqaciGacaGaaeqabaqabeGadaaakeaacqWG5bqEcqGGOaakcqWG0baDcqGGPaqkcqGH9aqpdaaeWbqaaiabdggaHnaaBaaaleaacqWGPbqAaeqaaOGaemOzay2aaSbaaSqaaiabdMgaPbqabaGccqGGOaakcqWG0baDcqGGPaqkaSqaaiabdMgaPjabg2da9iabicdaWaqaaiabd6eaojabgkHiTiabigdaXaqdcqGHris5aaaa@43CA@

where *f*_*i*_*(t) *is the electropherogram of *i*-th component, *t *– the scan number or time after the start of electrophoresis, *a*_*i *_– the quantity of the *i*-th component and *N *– number of components. Thus, the problem can be converged to finding *a*_*i*_, which is the best linear fit given that *f*_*i*_*(t) *is known beforehand – again from the set of pre-recorded electropherograms of expected components. One solves a system of linear equations:

**FA **= **Y**

where **A **is a {*a*_*i*_}^T ^vector, **F **– matrix, which *k*-th row is {*f*_*i*_*(t_*k*_)*} and **Y **– vector {*y(t_*k*_)*}^T^. The index *k *runs from 0 to at least *N*-1. The t_k _is a subset of scans from the entire electropherogram. The solution is found as shown above

The functions {*f*_*i*_*(t)*} are determined by sequencing the individual components and storing the electropherograms in the profile library.

#### Simulation of Dideoxy sequencing results

Gauss-shaped peaks were simulated for random sequences, with unequal assignment of peak height at each position to mimic the known differential incorporation effects. A component library was build from these simulated electropherograms. Concentrations of each component of the library were chosen to be 1.

A mixture was composed from randomized amounts of components adding up to 1. This way of mixture simulation reflects a realistic case where the total amount of DNA or RNA of the mix would be the same as that used to record the library. The distribution of the components was chosen to represent abundant and rare components (compare Fig. 3). The system was solved as described above. Solutions represent fractions of each component of the library in the sample mixture. To estimate the influence of noise, normally distributed random numbers were used to change each peak in the simulated electropherogram of the sample and the library at a specified noise level. The noise level was determined as a fraction of the maximal peak of an electropherogram.

## Authors' contributions

A.P. has developed the algorithm and conducted the simulation experiments, as well as initial experimental test. K.S. has done the pyrosequencing experiments. D.T. has initiated and devised the approach and has written the manuscript together with A.P.

**Figure 4:**
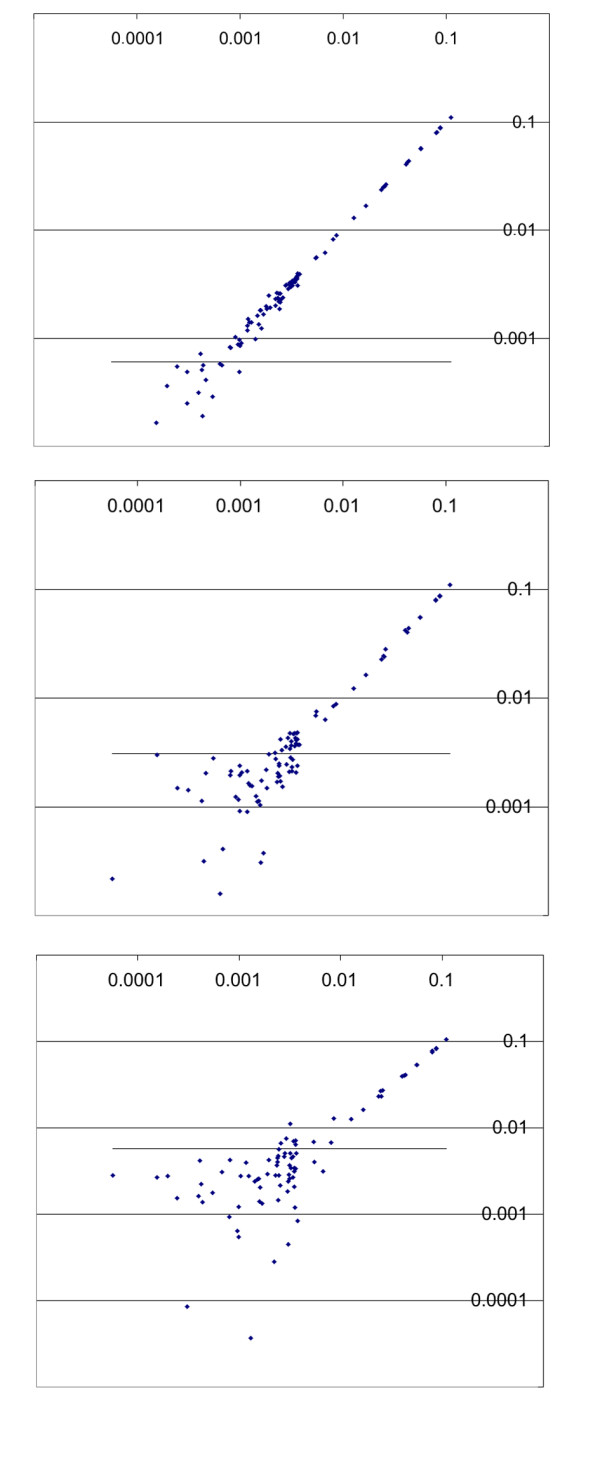
Simulation of the effect of noise on the recovery of the correct concentrations of the components in a mix of 99 samples with different concentrations. The distribution of the concentrations is shown in Figure 3. From top to bottom: 1, 5 and 10 % noise level. The graphs represent a direct comparison of given (X axis) and found (Y axis) values. A solid line indicates detection limit calculated as 3 times standard deviation of the negative control. Solutions below this line are not reliable.

## Supplementary Material

Additional File 1 Supplemetary Table 1 lists the individual values for the pyrosequencing deconvolution experiment Click here for file
