# Supplementary material for: An algorithm for the determination and quantification of components of nucleic acid mixtures based on single sequencing reactions
Source: BMC Bioinformatics. 2005 Nov 29;6:281. doi: 10.1186/1471-2105-6-281 (PMC1318471; doi:10.1186/1471-2105-6-281)
Supplement: Additional File 1 — Supplemetary Table 1 lists the individual values for the pyrosequencing deconvolution experiment [file 1471-2105-6-281-S1.pdf]

	observed average	SD of average	expected
**mix 1**			
**Algae**	0,004	0,01	0,00
**Cyclops**	0,204	0,01	0,17
**Ephemeroptera**	0,204	0,04	0,17
**Harpacticoid**	0,196	0,02	0,17
**Nematod**	0,100	0,01	0,17
**Ostracod**	0,177	0,02	0,17
**Tardigrade**	0,116	0,02	0,17
**mix 2**			
**Algae**	0,004	0,01	0,00
**Cyclops**	0,154	0,01	0,13
**Ephemeroptera**	0,064	0,02	0,06
**Harpacticoid**	0,331	0,02	0,31
**Nematod**	0,076	0,01	0,13
**Ostracod**	0,329	0,02	0,31
**Tardigrade**	0,042	0,01	0,06
**mix 3**			
**Algae**	-0,001	0,01	0,00
**Cyclops**	0,391	0,02	0,31
**Ephemeroptera**	0,169	0,03	0,13
**Harpacticoid**	0,092	0,01	0,06
**Nematod**	0,186	0,02	0,31
**Ostracod**	0,070	0,02	0,06
**Tardigrade**	0,093	0,01	0,13
**mix 4**			
**Algae**	0,003	0,01	0,00
**Cyclops**	0,107	0,04	0,06
**Ephemeroptera**	0,333	0,04	0,31
**Harpacticoid**	0,138	0,03	0,13
**Nematod**	0,031	0,01	0,06
**Ostracod**	0,155	0,03	0,13
**Tardigrade**	0,233	0,03	0,31

Averages and standard deviations for the replicates for each mix. Individual values are listed below.

mix 1: A : C : E : H : N : O : T = 0 : 0.17 : 0.17 : 0.17 : 0.17 : 0.17 : 0.17								
	values found				standard deviations			
replicates	1	2	3	4	1	2	3	4
library 1								
Algae	0,000	0,003	0,013	0,006	0,006	0,007	0,007	0,011
Cyclops	0,200	0,200	0,216	0,194	0,010	0,011	0,012	0,019
Ephemeroptera	0,177	0,189	0,169	0,208	0,011	0,011	0,012	0,019
Harpacticoid	0,203	0,212	0,187	0,207	0,011	0,012	0,013	0,020
Nematod	0,118	0,118	0,116	0,112	0,009	0,010	0,011	0,017
Ostracod	0,206	0,186	0,200	0,185	0,014	0,014	0,016	0,025
Tardigrade	0,095	0,093	0,099	0,087	0,004	0,004	0,004	0,006
library 2								
Algae	-0,008	0,002	0,014	0,000	0,011	0,013	0,010	0,021
Cyclops	0,212	0,210	0,227	0,202	0,011	0,014	0,010	0,021
Ephemeroptera	0,186	0,198	0,175	0,217	0,011	0,013	0,010	0,021
Harpacticoid	0,209	0,214	0,192	0,210	0,011	0,013	0,010	0,021
Nematod	0,104	0,106	0,100	0,101	0,009	0,011	0,008	0,017
Ostracod	0,178	0,154	0,170	0,163	0,013	0,016	0,012	0,024
Tardigrade	0,119	0,116	0,122	0,107	0,005	0,006	0,004	0,009
library 3								
Algae	-0,007	-0,001	0,015	0,001	0,010	0,010	0,011	0,016
Cyclops	0,205	0,204	0,220	0,193	0,011	0,011	0,012	0,018
Ephemeroptera	0,246	0,260	0,232	0,286	0,015	0,015	0,016	0,023
Harpacticoid	0,167	0,173	0,153	0,168	0,009	0,009	0,010	0,015
Nematod	0,085	0,084	0,081	0,080	0,007	0,007	0,007	0,011
Ostracod	0,170	0,150	0,161	0,152	0,011	0,012	0,013	0,018
Tardigrade	0,134	0,130	0,138	0,119	0,005	0,005	0,006	0,008
library 4								
Algae	0,000	0,005	0,015	0,007	0,007	0,008	0,009	0,014
Cyclops	0,192	0,193	0,207	0,188	0,009	0,010	0,011	0,017
Ephemeroptera	0,170	0,183	0,162	0,201	0,010	0,011	0,012	0,019
Harpacticoid	0,210	0,217	0,195	0,213	0,010	0,011	0,012	0,019
Nematod	0,099	0,100	0,096	0,094	0,008	0,009	0,010	0,015
Ostracod	0,200	0,177	0,193	0,181	0,013	0,015	0,016	0,024
Tardigrade	0,128	0,124	0,131	0,117	0,004	0,005	0,005	0,008

mix 2: A : C : E : H : N : O : T = 0 : 0.13 : 0.06 : 0.31 : 0.13 : 0,31 : 0.06								
	values found				standard deviations			
replicates	1	2	3	4	1	2	3	4
library 1								
Algae	0,014	-0,002	0,002	0,010	0,007	0,005	0,006	0,013
Cyclops	0,144	0,149	0,153	0,134	0,012	0,008	0,010	0,022
Ephemeroptera	0,034	0,049	0,058	0,069	0,012	0,008	0,010	0,023
Harpacticoid	0,339	0,316	0,331	0,331	0,013	0,009	0,011	0,024
Nematod	0,071	0,094	0,091	0,095	0,011	0,007	0,009	0,020
Ostracod	0,358	0,364	0,331	0,340	0,016	0,011	0,013	0,029
Tardigrade	0,040	0,030	0,035	0,021	0,004	0,003	0,003	0,008
library 2								
Algae	0,021	-0,014	-0,005	0,001	0,015	0,011	0,011	0,023
Cyclops	0,156	0,166	0,169	0,149	0,015	0,011	0,012	0,023
Ephemeroptera	0,041	0,060	0,069	0,079	0,014	0,011	0,011	0,023
Harpacticoid	0,360	0,339	0,352	0,349	0,014	0,011	0,011	0,023
Nematod	0,060	0,083	0,080	0,086	0,012	0,009	0,009	0,018
Ostracod	0,310	0,328	0,291	0,312	0,017	0,013	0,013	0,027
Tardigrade	0,052	0,038	0,044	0,024	0,006	0,005	0,005	0,010
library 3								
Algae	0,023	-0,009	-0,003	0,008	0,015	0,010	0,011	0,021
Cyclops	0,166	0,175	0,178	0,152	0,017	0,011	0,012	0,023
Ephemeroptera	0,056	0,082	0,095	0,116	0,022	0,014	0,016	0,030
Harpacticoid	0,316	0,297	0,306	0,308	0,014	0,009	0,010	0,019
Nematod	0,054	0,074	0,070	0,075	0,010	0,006	0,007	0,014
Ostracod	0,320	0,333	0,299	0,311	0,018	0,011	0,013	0,024
Tardigrade	0,065	0,048	0,056	0,031	0,008	0,005	0,006	0,011
library 4								
Algae	0,019	-0,004	0,001	0,009	0,011	0,007	0,008	0,015
Cyclops	0,139	0,148	0,152	0,136	0,014	0,008	0,011	0,019
Ephemeroptera	0,034	0,050	0,059	0,069	0,015	0,009	0,011	0,021
Harpacticoid	0,349	0,327	0,341	0,342	0,015	0,009	0,011	0,021
Nematod	0,054	0,076	0,074	0,075	0,012	0,007	0,009	0,017
Ostracod	0,347	0,359	0,324	0,339	0,020	0,012	0,015	0,027
Tardigrade	0,058	0,043	0,050	0,031	0,006	0,004	0,005	0,009

mix 3: A : C : E : H : N : O : T = 0 : 0.31 : 0.13 : 0.06 : 0.31 : 0.06 : 0.13								
	values found				standard deviations			
replicates	1	2	3	4	1	2	3	4
library 1								
Algae	0,000	0,008	-0,002	0,005	0,006	0,006	0,006	0,010
Cyclops	0,400	0,403	0,386	0,367	0,009	0,010	0,009	0,017
Ephemeroptera	0,156	0,151	0,137	0,170	0,010	0,010	0,009	0,017
Harpacticoid	0,077	0,094	0,087	0,102	0,010	0,010	0,010	0,018
Nematod	0,227	0,208	0,216	0,204	0,008	0,009	0,008	0,015
Ostracod	0,065	0,058	0,099	0,082	0,012	0,013	0,012	0,022
Tardigrade	0,075	0,078	0,076	0,070	0,003	0,003	0,003	0,006
library 2								
Algae	-0,008	0,006	-0,012	-0,001	0,010	0,010	0,008	0,019
Cyclops	0,417	0,416	0,403	0,381	0,010	0,010	0,008	0,019
Ephemeroptera	0,162	0,154	0,143	0,173	0,009	0,010	0,008	0,018
Harpacticoid	0,088	0,101	0,098	0,108	0,009	0,009	0,008	0,018
Nematod	0,200	0,184	0,191	0,182	0,008	0,008	0,006	0,015
Ostracod	0,049	0,043	0,082	0,072	0,011	0,011	0,009	0,022
Tardigrade	0,092	0,095	0,094	0,085	0,004	0,004	0,003	0,008
library 3								
Algae	-0,009	0,002	-0,010	-0,001	0,008	0,007	0,008	0,014
Cyclops	0,406	0,406	0,396	0,370	0,009	0,007	0,009	0,016
Ephemeroptera	0,217	0,209	0,192	0,235	0,012	0,010	0,012	0,021
Harpacticoid	0,064	0,077	0,074	0,084	0,007	0,006	0,007	0,013
Nematod	0,160	0,145	0,154	0,144	0,005	0,004	0,005	0,010
Ostracod	0,058	0,053	0,086	0,073	0,009	0,008	0,009	0,016
Tardigrade	0,105	0,108	0,108	0,096	0,004	0,003	0,004	0,007
library 4								
Algae	-0,002	0,008	-0,004	0,004	0,007	0,007	0,006	0,012
Cyclops	0,387	0,389	0,372	0,356	0,009	0,008	0,008	0,015
Ephemeroptera	0,156	0,149	0,135	0,167	0,010	0,009	0,008	0,016
Harpacticoid	0,091	0,105	0,101	0,114	0,010	0,009	0,008	0,016
Nematod	0,204	0,188	0,193	0,182	0,008	0,007	0,007	0,013
Ostracod	0,061	0,054	0,097	0,080	0,013	0,012	0,011	0,021
Tardigrade	0,104	0,107	0,105	0,097	0,004	0,004	0,004	0,007

mix4: A : C : E : H : N : O : T = 0 : 0.06 : 0.31 : 0.13 : 0.06 : 0.13 : 0.31								
	values found				standard deviations			
replicates	1	2	3	4	1	2	3	4
library 1								
Algae	-0,007	0,001	0,003	0,016	0,007	0,006	0,007	0,035
Cyclops	0,083	0,083	0,101	0,190	0,011	0,010	0,011	0,057
Ephemeroptera	0,329	0,334	0,337	0,285	0,011	0,010	0,012	0,058
Harpacticoid	0,178	0,156	0,156	0,093	0,012	0,011	0,012	0,061
Nematod	0,035	0,052	0,039	0,037	0,010	0,009	0,010	0,051
Ostracod	0,171	0,169	0,158	0,214	0,015	0,013	0,015	0,076
Tardigrade	0,211	0,203	0,205	0,166	0,004	0,003	0,004	0,020
library 2								
Algae	-0,015	-0,006	0,003	0,022	0,013	0,009	0,011	0,058
Cyclops	0,078	0,080	0,096	0,185	0,013	0,009	0,011	0,059
Ephemeroptera	0,332	0,336	0,335	0,288	0,013	0,009	0,011	0,058
Harpacticoid	0,171	0,151	0,152	0,105	0,012	0,008	0,011	0,057
Nematod	0,030	0,045	0,031	0,025	0,010	0,007	0,009	0,047
Ostracod	0,145	0,147	0,133	0,174	0,015	0,010	0,012	0,068
Tardigrade	0,258	0,247	0,250	0,201	0,005	0,004	0,004	0,024
library 3								
Algae	-0,015	-0,005	0,000	0,019	0,010	0,009	0,010	0,050
Cyclops	0,070	0,070	0,085	0,168	0,011	0,010	0,011	0,054
Ephemeroptera	0,402	0,407	0,405	0,358	0,015	0,013	0,015	0,072
Harpacticoid	0,131	0,115	0,114	0,074	0,009	0,008	0,009	0,045
Nematod	0,023	0,034	0,026	0,022	0,007	0,006	0,007	0,033
Ostracod	0,124	0,125	0,115	0,148	0,012	0,010	0,012	0,057
Tardigrade	0,264	0,254	0,255	0,210	0,005	0,004	0,005	0,025
library 4								
Algae	-0,003	0,004	0,009	0,024	0,011	0,009	0,010	0,042
Cyclops	0,077	0,077	0,091	0,176	0,014	0,012	0,012	0,053
Ephemeroptera	0,301	0,306	0,309	0,266	0,015	0,013	0,013	0,057
Harpacticoid	0,180	0,161	0,160	0,109	0,015	0,013	0,013	0,057
Nematod	0,020	0,035	0,024	0,015	0,012	0,010	0,011	0,047
Ostracod	0,158	0,160	0,147	0,193	0,019	0,017	0,017	0,075
Tardigrade	0,267	0,256	0,260	0,217	0,006	0,005	0,006	0,025
